# Glycosylation of Sodium/Iodide Symporter (NIS) Regulates Its Membrane Translocation and Radioiodine Uptake

**DOI:** 10.1371/journal.pone.0142984

**Published:** 2015-11-23

**Authors:** Taemoon Chung, Hyewon Youn, Chan Joo Yeom, Keon Wook Kang, June-Key Chung

**Affiliations:** 1 Department of Nuclear Medicine, Seoul National University College of Medicine, Seoul, Korea; 2 Biomedical Sciences, Seoul National University College of Medicine, Seoul, Korea; 3 Cancer Research Institute, Seoul National University College of Medicine, Seoul, Korea; 4 Tumor Microenvironment Global Core Research Center, Seoul National University, Seoul, Korea; 5 Cancer Imaging Center, Seoul National University Hospital, Seoul, Korea; ACTREC, Tata Memorial Centre, INDIA

## Abstract

**Purpose:**

Human sodium/iodide symporter (hNIS) protein is a membrane glycoprotein that transports iodide ions into thyroid cells. The function of this membrane protein is closely regulated by post-translational glycosylation. In this study, we measured glycosylation-mediated changes in subcellular location of hNIS and its function of iodine uptake.

**Methods:**

HeLa cells were stably transfected with hNIS/tdTomato fusion gene in order to monitor the expression of hNIS. Cellular localization of hNIS was visualized by confocal microscopy of the red fluorescence of tdTomato. The expression of hNIS was evaluated by RT-PCR and immunoblot analysis. Functional activity of hNIS was estimated by radioiodine uptake. Cyclic AMP (cAMP) and tunicamycin were used to stimulate and inhibit glycosylation, respectively. In vivo images were obtained using a Maestro fluorescence imaging system.

**Results:**

cAMP-mediated Glycosylation of NIS resulted in increased expression of hNIS, stimulating membrane translocation, and enhanced radioiodine uptake. In contrast, inhibition of glycosylation by treatment with tunicamycin dramatically reduced membrane translocation of intracellular hNIS, resulting in reduced radioiodine uptake. In addition, our hNIS/tdTomato fusion reporter successfully visualized cAMP-induced hNIS expression in xenografted tumors from mouse model.

**Conclusions:**

These findings clearly reveal that the membrane localization of hNIS and its function of iodine uptake are glycosylation-dependent, as our results highlight enhancement of NIS expression and glycosylation with subsequent membrane localization after cAMP treatment. Therefore, enhancing functional NIS by the increasing level of glycosylation may be suggested as a promising therapeutic strategy for cancer patients who show refractory response to conventional radioiodine treatment.

## Introduction

Sodium/iodide symporter (NIS) is a membrane glycoprotein expressed on the plasma membrane of thyroid follicular cells. NIS actively transports iodine ions to thyroid follicular cells and is essential for thyroid hormone synthesis [1,2]. The persistent expression of NIS in differentiated thyroid carcinoma (DTC) induces iodine accumulation in thyroid cancer cells. As a result, radioiodine whole body scintigraphy and radioiodine therapy have been widely used in the management of DTC patients [3,4]. It is investigated that advantages of NIS as a reporter gene are non-immunogenic with no observed adverse effects on function and cell viability [5]. To take advantage of diagnostic and therapeutic use of NIS, there are ongoing studies for imaging and treating thyroid cancer, as well as other types of cancer that do not express NIS [6,7]. After incorporating NIS gene into various human cancer cells as a therapeutic gene, various radionuclides such as ^188^Re and ^131^I were used to assess the therapeutic effect in the preclinical stage [8–10]. Also, there are studies exploring the feasibility of using NIS gene as a reporter for in vivo imaging [11–13].

NIS has 13 transmembrane segments with three putative *N-*linked glycosylation sites (Asn485, Asn497, and Asn225 in case of rat NIS) [14,15]. Membrane protein is generally produced in the endoplasmic reticulum, and then move to cellular membrane through the golgi complex. This membrane localization process requires proper protein post-translational modification including phosphorylation and glycosylation [16,17]. Levi *et al*. have observed that NIS is highly glycosylated in membranes [18], and glycosylation is somehow related to functional maturation of a membrane protein and its trans-localization to plasma membrane [19]. Regulation of glycosylation can be tested with activators and inhibitors. Cyclic adenosine monophosphate (cAMP) has been shown to increase N-glycosylation capacity due to enlargement of the dolichol pyrophosphoryl oligosaccharide [20–22]. In contrast, tunicamycin completely blocks protein glycosylation by inhibition of the formation of N-acetylglucosamine-lipid intermediates [23–26], which results in decreased activity of membrane transporters because the membrane proteins remain localized in intracellular compartments due to decreased translocation to the cell surface membrane [27]. It has been reported that down-regulation of glycosylation of NIS protein decreased function of NIS protein [28]. However, visualization of the intracellular localization of NIS protein affected by regulation of glycosylation has not been reported.

The subcellular expression and localization of membrane proteins can easily be visualized by gene fusion with a fluorescent protein [29]. To analyze the effect of glycosylation regulation on hNIS cellular localization, we utilized a gene fusion system with hNIS and red fluorescent protein expressing tandem dimeric Tomato (tdTomato), which was derived by several mutations of DsRed to improve brightness and photostability [30,31]. Using hNIS/tdTomato, we measured glycosylation-mediated changes in subcellular location of hNIS and its function of iodine uptake.

## Materials and Methods

### Cells

HeLa cells (a human cervical epithelial adenocarcinoma cell line, ATCC, Manassas, VA, USA) were maintained in Dulbecco’s modified Eagle’s medium (DMEM, Gibco-Invitrogen, Eugene, OR, USA) supplemented with 10% fetal bovine serum and 1% antibiotic-antimycotic solution (Gibco-Invitrogen, Eugene, OR, USA) at 37°C in a 5% CO_2_ humidified atmosphere. Cells were transfected with pCMV-hNIS/tdTomato using Lipofectamine-plus (Invitrogen, Carlsbad, CA, USA) according to the manufacturer’s instructions. Stably transfected cells were selected in medium containing 1,000 μg/mL of geneticin (Gibco-Invitrogen, Eugene, OR, USA) for 2 weeks.

### Regulation of glycosylation

For regulation of glycosylation, HeLa-hNIS/tdTomato cells were incubated in a 24 well plate with an inhibitor or activator for 24 to 72 hr (h). To inhibit glycosylation, cells were treated with tunicamycin (Sigma-Aldrich, St. Louis, MO, USA) at 0.3, 0.6, or 1.2 μM. To stimulate glycosylation, cells were treated with cAMP (Sigma-Aldrich, St. Louis, MO, USA) at 10, 50, 100, or 1000 μM. To inhibit transcriptional or translational regulation of cAMP induced-hNIS expression, HeLa-hNIS/tdTomato cells were treated with 5 ng/mL of actinomycin D (AMD, Sigma-Aldrich, St. Louis, MO, USA) or 1 μg/ml of cycloheximide (CHX, Sigma-Aldrich, St. Louis, MO, USA) for 24 hr before cAMP treatment.

### RT-PCR

Total RNA was extracted with TRIzol reagent (Invitrogen, Carlsbad, CA, USA) according to the manufacturer’s protocol. Reverse-transcription was performed with 1 μg of total RNA using a cDNA synthesis master kit (Genedepot, Houaron, TX, USA). PCR was performed to quantify the expression of hNIS and tdTomato genes in hNIS/tdTomato transfected cells. The primer pairs used were as follows: hNIS forward 5´-ACTTTGCAGTACATTGTAGCC-3´ and reverse 5´-ACAGTGACTGCAGCCATAG-3´; tdTomato forward 5´-CAAGGGCGAGGAGGTCAT-3´ and reverse 5´-GTGCTGCCGGTGCCATG-3´; and β-actin (internal control) forward 5´TGACGGGGTCACCCAACTGTGCCCATCTA-3´ and reverse 5´-CTAGAAGCATTTGCGGTGGACGATGGAGGG-3´. PCR conditions were 60 sec at 94°C for denaturation, 40 sec at 57°C for primer annealing, and 40 sec at 72°C for primer extension. PCR products were analyzed by 1.2% agarose gel electrophoresis.

### Immunoblot analysis

HeLa-hNIS/tdTomato cell lysates were isolated using RIPA protein lysis buffer. Membrane protein fractions were isolated using a Mem-PER eukaryotic membrane protein extraction reagent kit (Pierce Biotech, Rockford, IL, USA) according to the manufacturer’s instructions. Protein concentrations were measured using a BCA Protein Assay Kit (Pierce Biotech, Rockford, IL, USA). Protein samples (20 μg) were mixed with a sodium dodecyl sulfate sample buffer, boiled at 95°C for 5 min, and separated using polyacrylamide gel electrophoresis (Invitrogen, Carlsbad, CA, USA). After proteins were transferred to a polyvinylidene difluoride membrane (PVDF, Millipore, Billerica, MA, USA), membranes were blocked with 5% skim milk in Tris-buffered saline containing Tween-20 (TBS-T, 20 mM Tris, 137 mM sodium chloride, and 0.1% Tween 20) for 1 h at room temperature. Then, membranes were incubated with antibodies specific to hNIS (1:2000, Koma biotech, Seoul, Korea), tdTomato (1:2000, Clontech, Mountain view, CA, USA), or β-actin (1:5000, Sigma-Aldrich, St. Louis, MO, USA). Membranes were washed three times with TBS-T and incubated with a secondary anti-rabbit horseradish peroxidase-conjugated antibody (1:2,000 dilution) for 1.5 hr. Immuno-reactive bands were visualized using enhanced chemiluminescence reagents (Roche, Indianapolis, IN, USA) and detected with the LAS-3000 system (Fuji Film, Stockholm, Sweden).

### *In vitro* ^125^I uptake assay

^125^I uptake assay of HeLa-hNIS/tdTomato cells was determined as previously described [32]. HeLa-hNIS/tdTomato cells (1 × 10^5^) were serially diluted and seeded in 24-well plates. After 24 hr incubation at 37°C, plates were aspirated. Then, cells in each well were treated with 500 μL of Hank’s balanced salt solution (HBSS, Gibco-Invitrogen, Grand island, NY, USA), which contained 10 μM NaI, carrier-free Na^125^I with a specific activity of 3.7 kBq (0.1 μCi), 0.5% bovine serum albumin, and 10 mM 2-[4-(2-hydroxyethyl)-1-piperzinyl]ethanesulfonic acid-NaOH (pH 7.3) at 37°C for 30 min. The cells were then washed twice with 1 mL of an ice-cold, iodide-free HBSS buffer and detached with 0.1% SDS. Radioactivity was measured using a Cobra II gamma counter (Canberra Packard, Ontario, Canada). Uptake values were normalized to the amount of protein. Protein was measured using the BCA protein assay (Pierce Biotech, Rockford, IL, USA).

### Confocal microscopy

Subcellular localization of hNIS/tdTomato protein and its fluorescence intensity were determined by confocal microscopy using a LSM510 META confocal microscope with a 40x objective lens (Carl Zeiss Inc., Oberkochen, Germany). Excitation light was generated by diode and a helium-neon laser (405 nm and 543 nm). Cells were fixed for 10 min with 3.7% paraformaldehyde (USB, Cleveland, OH, USA), washed twice with PBS, and mounted with ProLong Gold antifade reagent with DAPI (Invitrogen, Carlsbad, CA, USA). Confocal microscope images were sectioned by Z-stacks at 1-μm-thick and retrieved by Zeiss LSM Imaging Browser version 3.5 (Carl Zeiss Inc.). MetaMorph software version 7.5.6 (MDS Analytical Technologies, Sunnyvale, CA, USA) was used for analysis of retrieved confocal microscope images. Threshold values of fluorescent signals by the MetaMorph “threshold” tool were used to determine the cytosolic and membrane portions of the cells as described previously [33].

### Tumor xenograft imaging in vivo

BALB/c nude mice (male, 6 to 8 weeks old) were used in accordance with the Institutional Animal Care and Use Committee of Seoul National University Hospital guidelines. HeLa-hNIS/tdTomato cells were incubated with 100 μM cAMP for 72 hr and 1 × 10^6^ cells were transplanted to the right flanks of mice. The same numbers of untreated HeLa-hNIS/tdTomato cells were transplanted to the left flanks of mice. In vivo fluorescence of hNIS/tdTomato was imaged using Maestro spectral fluorescence imaging system (Cambridge Research & Instrumentation, Inc., Woburn, MA, USA) and quantified using Maestro version 2.2 software as previously described [34]. Regions of interest (ROIs) were drawn on each fluorescent signal to quantify the total fluorescent signal.

### Statistical Analysis

All experiments were conducted at least in triplicate. Statistically significant differences between groups were determined using Student’s *t* test. All statistical analyses were performed using Microsoft Excel 2010. *P* value less than 0.05 was considered significant.

## Results and Discussion

### Imaging of hNIS expression in HeLa cells

**Fig 1:**
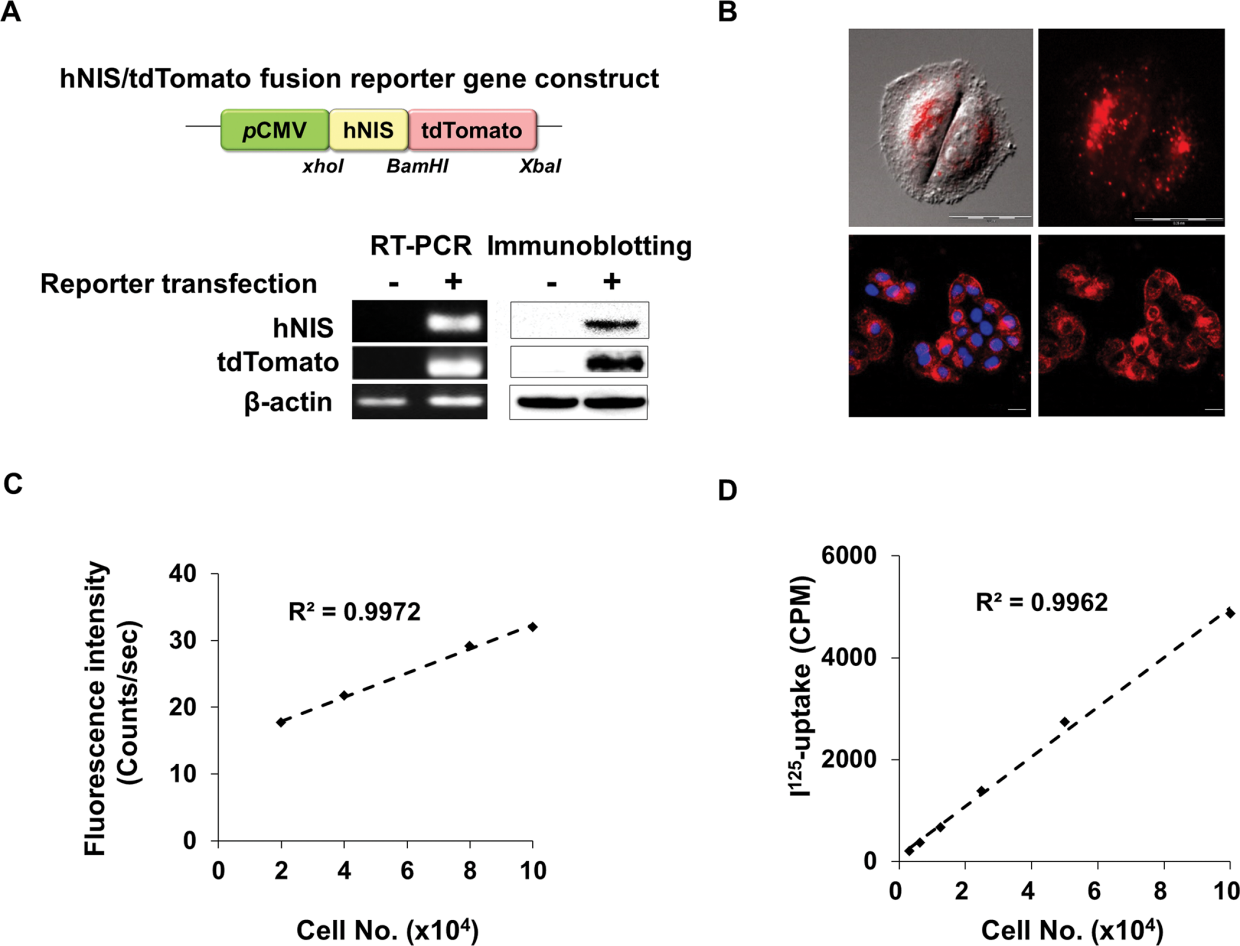
Generation of HeLa cells expressing the hNIS/tdTomato fusion gene. (A) Schematic representation of the hNIS/tdTomato fusion gene reporter construct (upper). RT-PCR (lower left) and Immunoblot analysis (lower right) showed stable expression of hNIS/tdTomato gene in HeLa cells. (B) Fluorescence microscope image shows the hNIS/tdTomato fusion protein expression in HeLa-hNIS/tdTomato cells. Cellular hNIS proteins were imaged using a time-lapse live cell imaging system (upper) and confocal microscopy (lower). (C) Red fluorescence from HeLa-hNIS/tdTomato cells increased with increasing cell number. (D) Function of hNIS/tdTomato in HeLa cells was measured by I-uptake and iodine uptake shows cell number dependency.

In order to monitor the cellular localization of the hNIS protein, we constructed a fusion reporter plasmid named hNIS/tdTomato that contains a red fluorescence protein-conjugated hNIS gene (Fig 1A, upper). We transfected the hNIS/tdTomato constructs into HeLa cells and isolated stably transfected cells with gentamycin. Selected HeLa-hNIS/tdTomato cells stably expressed hNIS and red fluorescence at both the mRNA and protein levels (Fig 1A, lower). Cellular hNIS protein expression was imaged using a time-lapse live cell imaging system (Fig 1B, upper) and confocal microscopy (Fig 1B, lower). The intra-cellular expression of hNIS proteins in HeLa-hNIS/tdTomato cells was visualized as red fluorescent dots. The signal intensity of red fluorescence and ^125^I-uptake in HeLa-hNIS/tdTomato cells were strongly correlated with the number of cells (Fig 1C and 1D). These results indicate that a hNIS/tdTomato fusion gene was successfully transfected into HeLa cells, and functional hNIS protein was produced in the HeLa-hNIS/tdTomato cells.

### Iodine uptake in HeLa-hNIS/tdTomato cells is regulated by glycosylation

Most membrane proteins, including hNIS, are produced in the cytosol and move to the cellular membrane. This membrane translocation process requires proper post-translational modifications including protein folding and glycosylation. For efficient iodine uptake, membrane localization of hNIS protein is essential. To investigate the effect of glycosylation on the function of hNIS, ^125^I-uptake by HeLa-hNIS/tdTomato cells was examined after treatment with tunicamycin or cAMP, which has known to regulate glycosylation of proteins.

**Fig 2:**
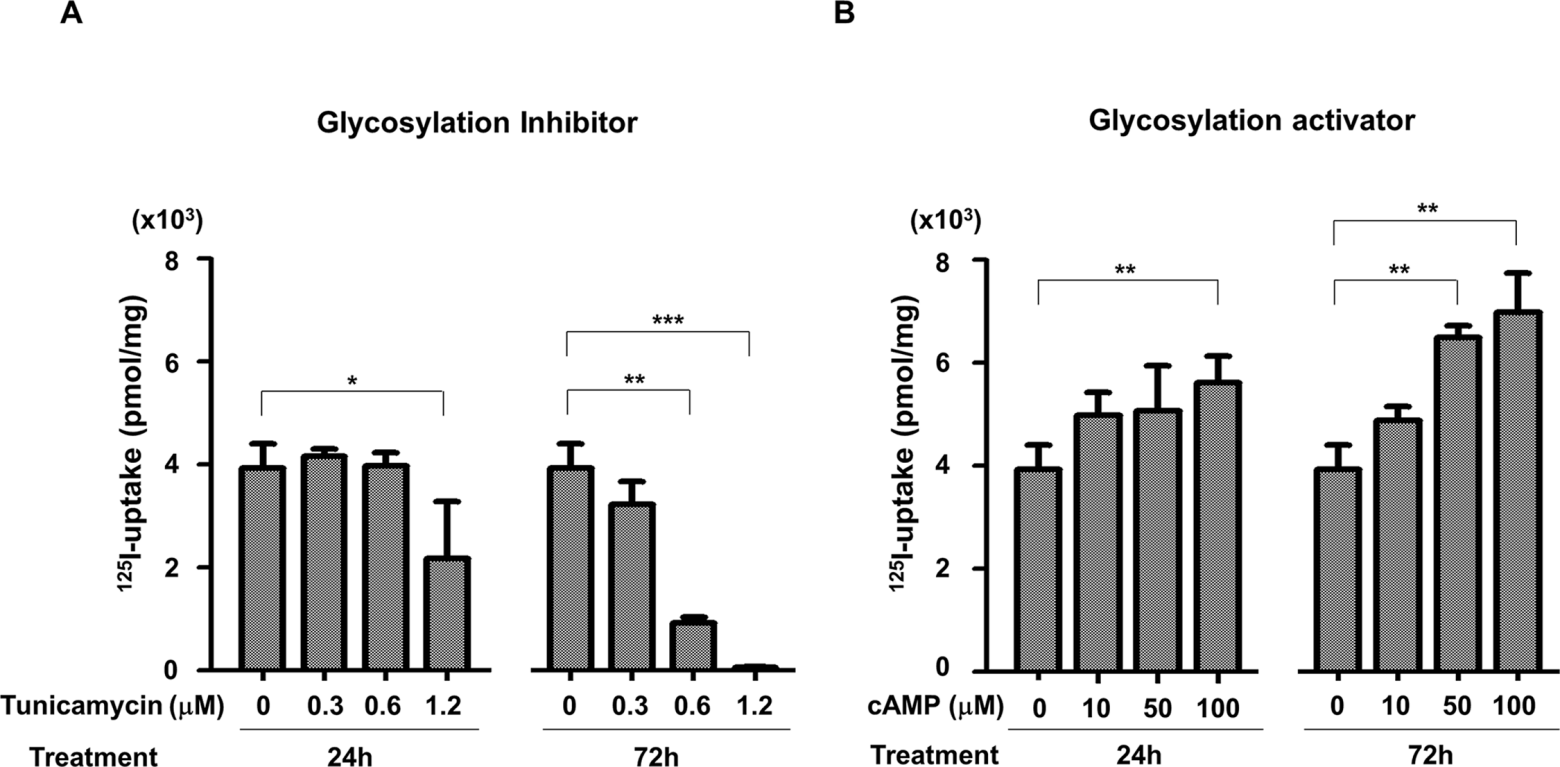
Changes of radioiodine uptake by regulation of glycosylation. (A) Glycosylation inhibitor, tunicamycin reduced radioiodine (^125^I) uptake in the hNIS/tdTomato expressing cells. (B) Glycosylation activator, cAMP increased radioiodine (^125^I) uptake in the hNIS/tdTomato expressing cells. Accumulation of radioiodine was measured with a gamma-counter at 24 hr and 72 hr after treatment (*, P<0.05; **, P<0.01; ***, P<0.001; N = 3).

Radioiodine uptake was decreased or increased with dose-dependent manner by treatment of tunicamycin or cAMP. Cells treated with 1.2 μM of tunicamycin for 24 hr showed 45% reduction of radioiodine uptake compared to untreated cells (Fig 2A, left). After 72 hr treatment of 1.2 μM tunicamycin, cells showed 90% reduction of radioiodine uptake (Fig 2A, right). In contrast, radioiodine uptake was increased to 42.6% after 24 hr incubation (Fig 2B, left) and 77.3% after 72 hr treatment with 100 μM of cAMP (Fig 2B, right) compared to control. These results revealed that hNIS function was strongly affected by regulation of glycosylation.

### Changes in sub-cellular localization of hNIS/tdTomato protein by regulation of glycosylation

**Fig 3:**
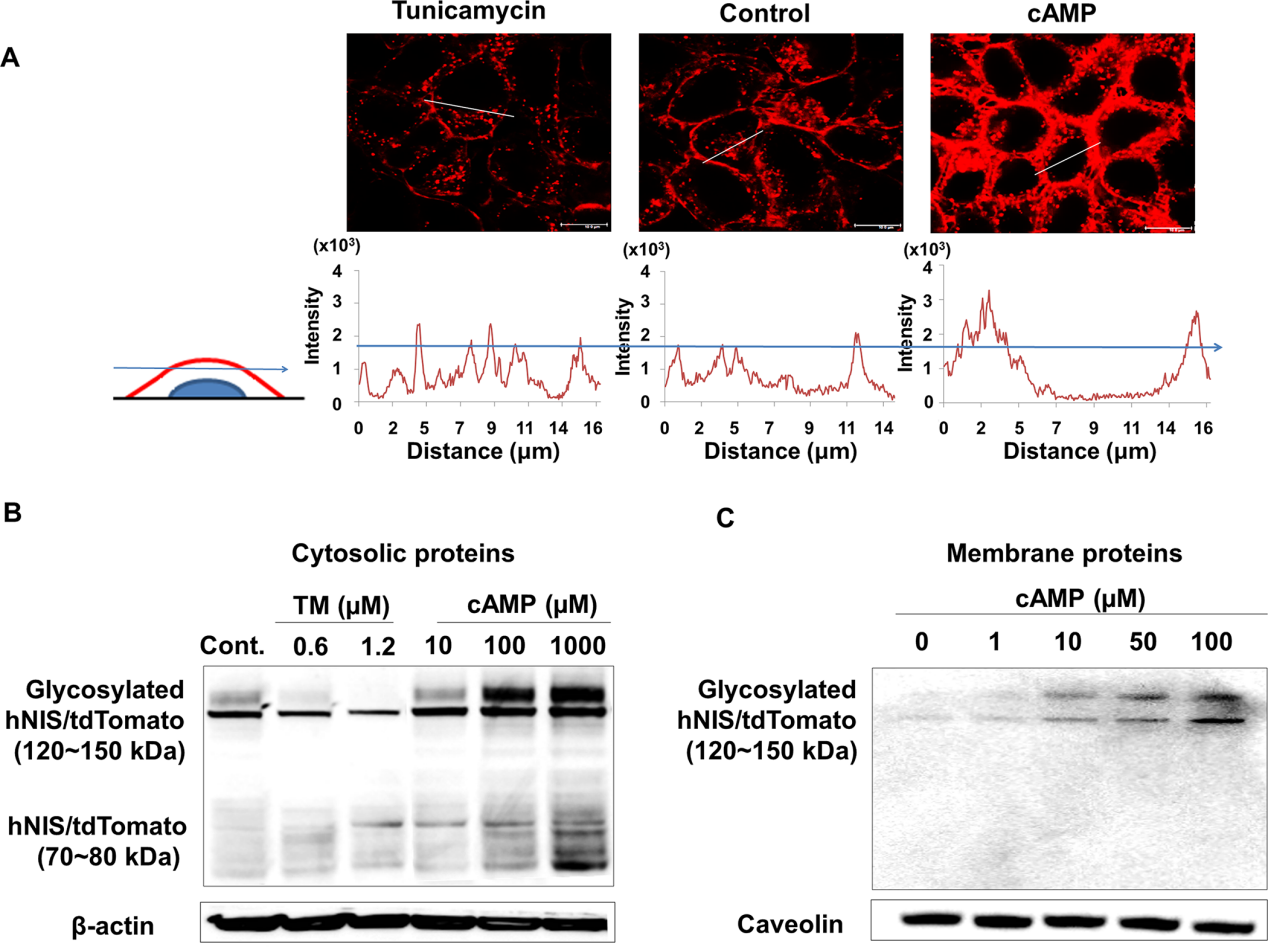
Membrane localization of glycosylated hNIS/tdTomato protein. (A) HeLa-hNIS/tdTomato cells were treated with tunicamycin (1.2 μM) or cAMP (100 μM), and the red fluorescent signals were photographed using confocal microscopy. Based on a cross-sectional analysis using fluorescence profiling of MetaMorph software, an arbitrary threshold that represented the cytosolic compartment was designated. Signals over the threshold were considered to be from the membrane compartment. (B) NIS expression was observed by immunoblot analysis with cellular protein extracts (20 μg) from tunicamycin- and cAMP-treated HeLa-hNIS/tdTomato cells. β-actin was used as an internal control. (C) NIS expression was observed by immunoblot analysis with membrane proteins isolated from cAMP-treated cells. Caveolin was used as an internal control.

To investigate changes in the intra-cellular localization of the hNIS protein by regulation of glycosylation, we analyzed cross-sectional confocal images of HeLa-hNIS/tdTomato cells after tunicamycin or cAMP treatment. To distinguish between fluorescent signals of hNIS/TdTomato protein in the cytosolic and membrane compartments, the cross-sectional analysis using fluorescence intensity profiling was used [33]. Fluorescence intensity profiles revealed that most of the hNIS/tdTomato protein was located in the cytosol after 72 hr treatment with tunicamycin (1.2 μM) to inhibit glycosylation (Fig 3A, left). In contrast, hNIS/tdTomato proteins were localized in the cell membrane as well as in the cytosol after treatment with cAMP (100 μM, Fig 3A, right). In addition, immunoblot analysis of cytosolic (Fig 3B) and membrane (Fig 3C) proteins from HeLa-NIS/tdTomato cells revealed that the glycosylated forms of cytosolic hNIS/tdTomato protein (120~150 kDa) [1,28,35] were significantly increased by cAMP treatment and decreased by tunicamycin treatment (Fig 3B). Especially, non-glycosylated form of hNIS/tdTomato (70~80 kDa) was present in the cytosolic compartment but not in the membrane compartment (Fig 3C). Therefore, it seems reasonable to assume that the elevated level of glycosylated hNIS protein may increase the amount of membrane-localized hNIS protein. The cytosolic level of 70~80 kDa hNIS/tdTomato did not change significantly following tunicamycin treatment (Fig 3B). Thus, it is likely that radioiodine uptake of hNIS increased after cAMP treatment due to an increased amount of membrane localized hNIS. These results indicate that regulation of glycosylation could change the level of hNIS protein in the membrane, which impacts iodine uptake.

Since cAMP is known to activate N-linked glycosylation, which leads to an increased transfer rate of the core oligosaccharide to target proteins [20], cAMP treatments seems to enhance membrane localization of hNIS. Tunicamycin blocks the addition of *N*-linked carbohydrates to the core protein and inhibits N-linked glycosylation [27]. When glycosylation is inhibited by tunicamycin, trafficking of the membrane protein to the cell surface is impaired [19], and proper membrane protein function seems to be disturbed. Like other membrane proteins, membrane localization of NIS proteins and their function were affected by regulation of glycosylation. It is reported that mutation of glycosylation site on NIS protein decreased function of iodide-uptake (50 to 90%) [28]. In addition, less glycosylated NIS showed reduced amount of membrane localized NIS proteins compare to fully glycosylated NIS [36]. These reports are in agreement with our results which showed modulation of glycosylation changed the membrane localization of NIS.

### Visualization and quantification of membrane-localized hNIS protein after regulation of glycosylation

**Fig 4:**
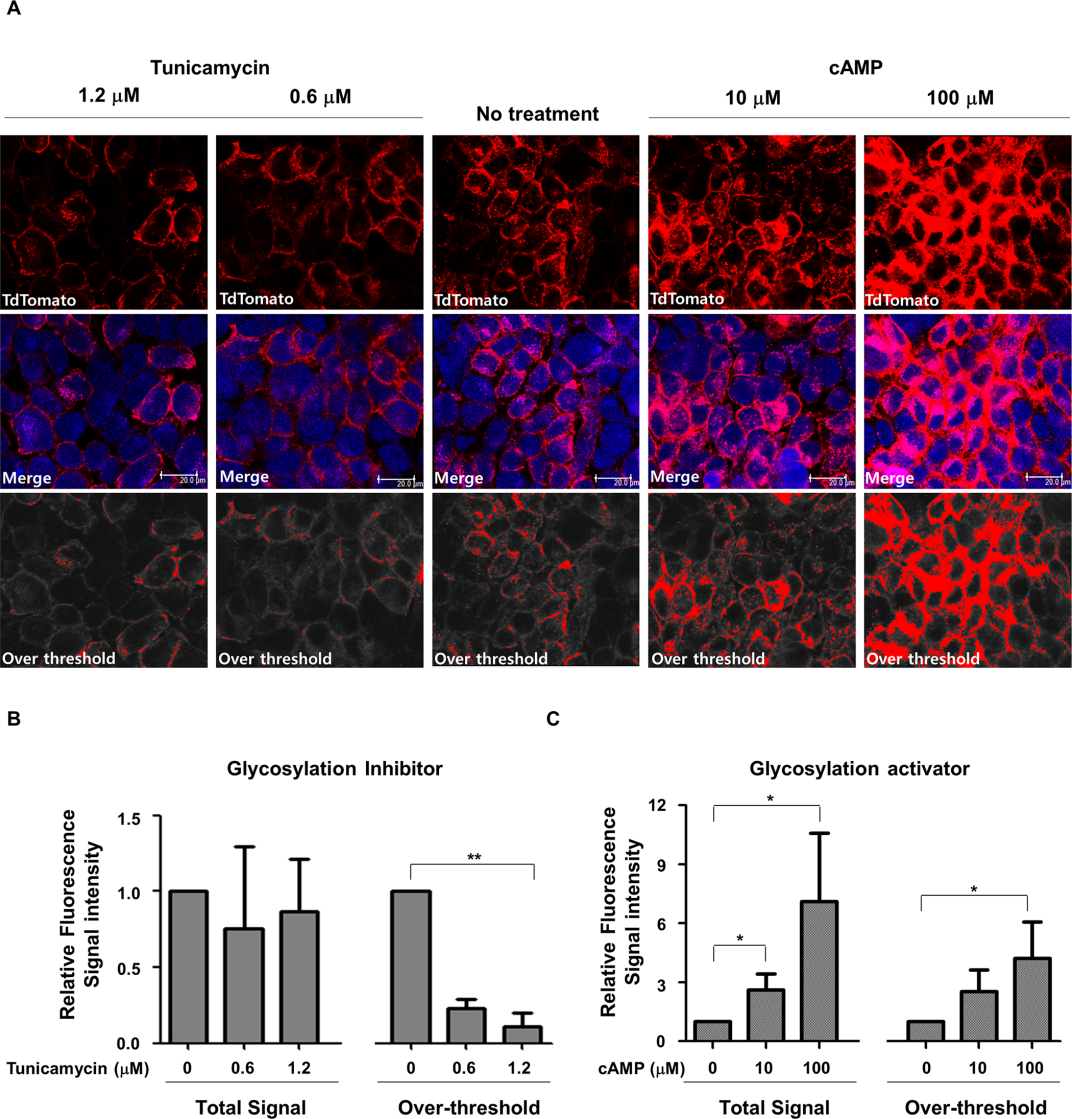
Visualization and image-based quantification of membrane-localized hNIS/tdTomato protein. (A) HeLa-hNIS/tdTomato cells were treated with tunicamycin or cAMP at the indicated concentrations, and red fluorescent signals were photographed using confocal microscopy. Based on a cross-sectional analysis using fluorescence profiling of MetaMorph software, an arbitrary threshold that represented the cytosolic compartment was designated. Signals over or under the threshold were depicted as red or gray, respectively. At least three different regions of each sample were imaged. Confocal images were collected from at least three different regions of each sample. (B) Relative fluorescence signal intensities of hNIS/tdTomato proteins after the treatment of glycosylation inhibitor (tunicamycin). (C) Relative fluorescence signal intensities of hNIS/tdTomato proteins after the treatment of glycosylation activator (cAMP). Fluorescent signal intensities acquired from threshold images were measured for quantification of membrane-localized hNIS/tdTomato proteins after glycosylation inhibitor or activator treatment. Relative fluorescence signal intensities were calculated based on the fluorescence intensity of non-treated control. Bars represent mean ± SD (*, P<0.05; **, P<0.01; N = 3).

To image-based quantification of the effect of glycosylation on the cellular localization of hNIS, we quantified the optical intensity in HeLa-hNIS/tdTomato cells using confocal microscopy. Total fluorescent signals from confocal microscope images were captured (Fig 4A, upper). An arbitrary threshold for the cytosolic compartment was obtained by integrating the signals from 1-μm-thick Z-stack cross-sections [34]. For normalization, the MetaMorph “manually count objects” tool was used to count the number of DAPI-stained nuclei and their intensity. Using the normalized fluorescent intensity value, stronger fluorescent signals were distinguished from weaker signals. While we designated the threshold for the cytosolic compartment, the over-threshold intensity may partially represent membrane-localized hNIS/tdTomato proteins. Based on a cross-sectional analysis using fluorescence intensity profiling, the over-threshold of red fluorescent intensity was calculated to quantify the membrane localized hNIS/tdTomato protein. After the integrated fluorescent intensities of the threshold images were measured, over- or under-threshold signals were constructed as red or gray, respectively (Fig 4A, lower).

The fluorescence in tunicamycin-treated cells occurred mainly in the cytosol (Fig 4A, left), and the level of protein expression did not change significantly (Fig 4B, left, total signal). However, the over-threshold signal showed a significant reduction in proportion to the tunicamycin concentration. Taken together, these results indicate that tunicamycin did not affect total hNIS/tdTomato protein expression but did affect its membrane localization. In addition, we observed that cAMP-treated cells exhibited strong cytosolic and membrane-localized fluorescent signals compared to untreated cells. Treatment with 100 μM of cAMP induced a 7-fold increase in the total signal and 5-fold increase in the over threshold signal of hNIS/tdTomato expression in HeLa-hNIS/tdTomato cells compared to untreated cells (Fig 4A, right and 4C). These results indicate that amount of the membrane-translocated hNIS proteins were altered by glycosylation-regulating agents.

### Membrane translocation of hNIS protein by cAMP after inhibition of de novo protein synthesis

In general hNIS expression is dependent on thyroid stimulation hormone (TSH, thyrotropin) and activated by cAMP via the cAMP response element (CRE) in the NIS upstream enhancer (NUE) region of the NIS promoter [22,37]. Instead of using the NIS promoter, we used a murine CMV promoter in this experiment for ectopic expression of hNIS/tdTomato without TSH. However, the CMV promoter also activated by cAMP [38,39]. Therefore, the localization of hNIS/tdTomato should be measured in the absence of cAMP-induced de novo hNIS protein synthesis. Pretreatment with 5 ng/mL of Actinomycin D (AMD) or 1 μg/ml of Cycloheximide (CHX) could efficiently block protein synthesis at the post transcriptional level (AMD) or translational level (CHX). Blocking protein synthesis using AMD or CHX, we could monitor the membrane translocation of pre-existed hNIS/tdtomato protein by cAMP without disturbance by newly synthesized hNIS/tdTomato protein.

**Fig 5:**
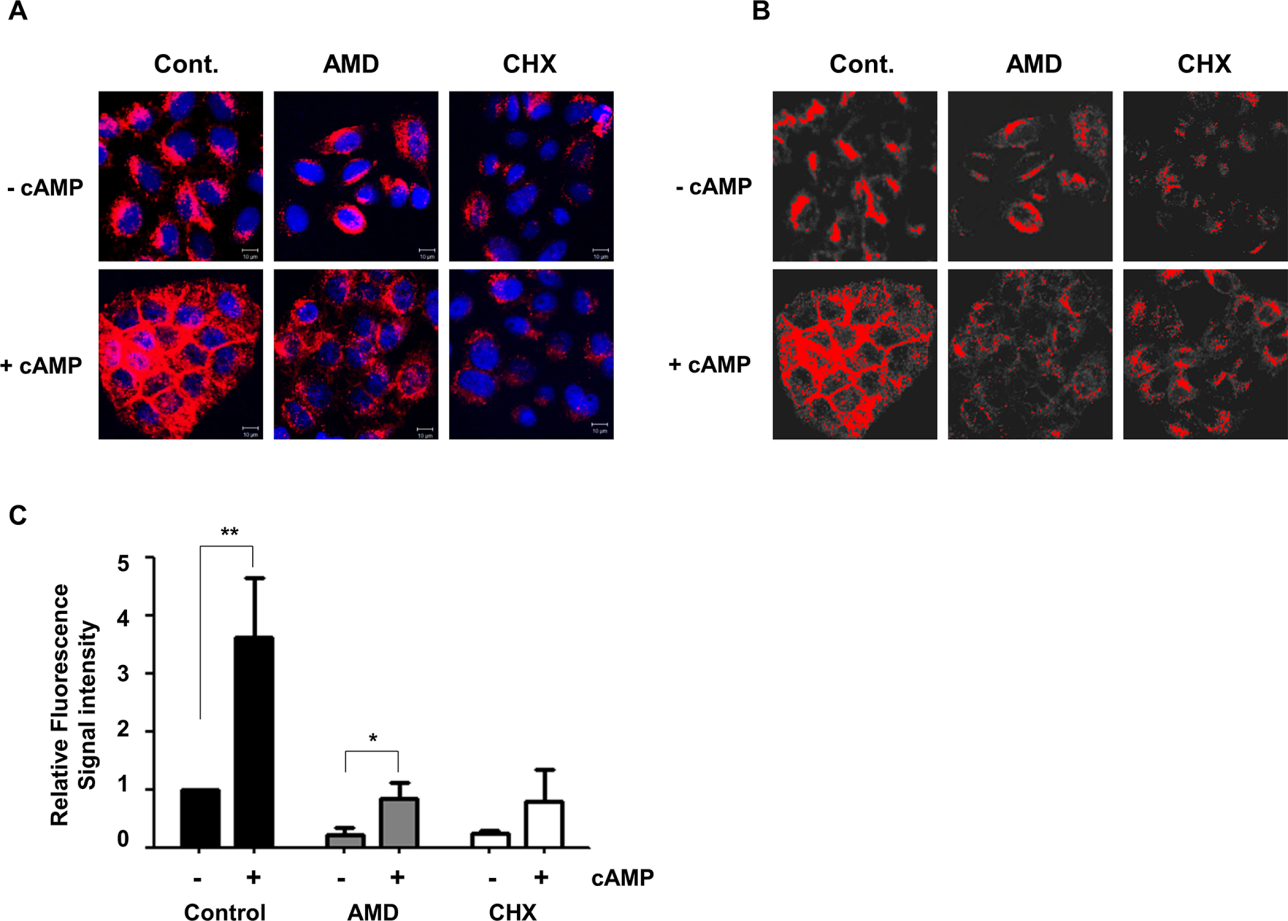
Membrane translocation of hNIS protein by cAMP after inhibition of de novo protein synthesis. To inhibit cAMP-induced protein synthesis, AMD (5 ng/mL) or CHX (1 μg/ml) were pretreated 24h before treatment of 100 μM cAMP. (A) Enhanced expression of hNIS/tdTomato proteins by cAMP was visualized after blocking de novo protein synthesis. (B) Enhanced membrane localization of hNIS/tdTomato proteins by cAMP was visualized after blocking de novo protein synthesis. Red fluorescent intensity was analyzed with MetaMorph software. An arbitrary threshold that represented the cytosolic compartment was designated. Threshold intensity of fluorescence was adjusted to show membrane-localized hNIS/tdTomato protein only. Signals over or under the threshold were depicted as red or gray, respectively. (C) The upper threshold of red fluorescent intensity was measured to quantify the membrane localized hNIS/tdTomato protein. Confocal images were collected from at least three different regions of each sample. Bars represent mean ± SD (*, P<0.05; **, P<0.01; N = 3).

As we expected, we found that cAMP enhances translocation of hNIS/tdTomato to the plasma membrane even after inhibition of de novo synthesis (Fig 5). Over-thereshold analysis clearly demonstrated that cAMP enhances membrane translocation of pre-existing hNIS/tdTomato to the plasma membrane. This indicates that cAMP not only increased hNIS production but also its membrane translocation by up-regulation of glycosylation.

### Enhancement of red fluorescence from cAMP-treated HeLa-hNIS/tdTomato cells in mouse

**Fig 6:**
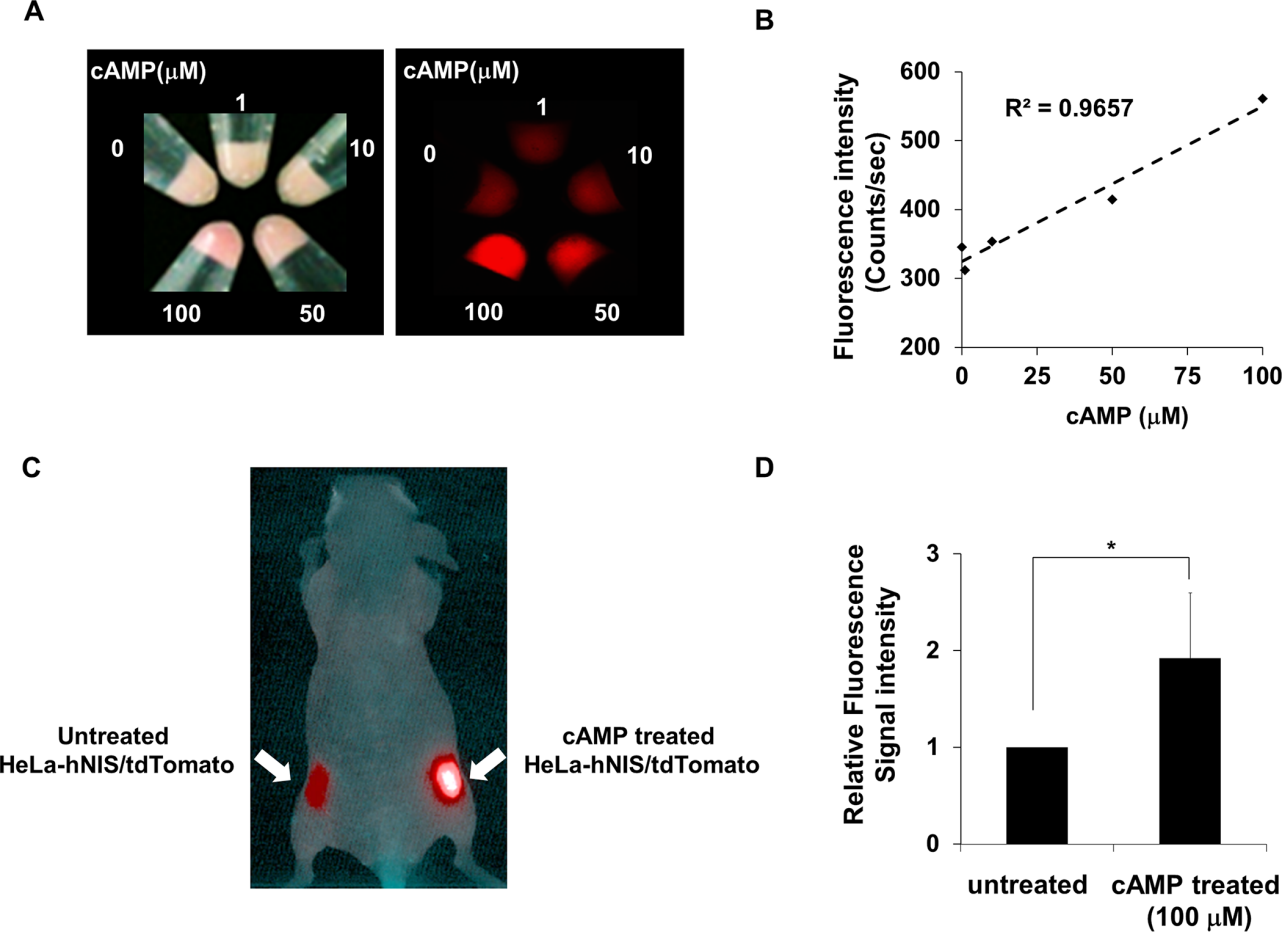
Enhancement of red fluorescence from cAMP treated HeLa-hNIS/tdTomato cells in mouse. (A) HeLa-hNIS/tdTomato cells (1x10^5^) were treated with cAMP (0–100 μM) at the indicated concentrations. Red fluorescent signals of cell pellets were imaged and measured using Maestro fluorescence imaging system. (B) The red fluorescent intensity that represents the expression of hNIS/tdTomato protein increased in proportion to the concentration of cAMP. (C) HeLa-hNIS/tdTomato cells were cultured with cAMP for 72 hr and then transplanted into the right flanks of mice. Untreated HeLa-hNIS/tdTomato cells were transplanted into the left flanks of mice. Red fluorescence of HeLa-hNIS/tdTomato was imaged by Maestro^TM^. (D) Red fluorescent intensity of HeLa-hNIS/tdTomato cells was analyzed by the Maestro^TM^ software program. Bars represent mean ± SD (*, P<0.05; N = 3).

The red fluorescent intensity that represents the expression of hNIS/tdTomato protein increased in proportion to the concentration of cAMP (Fig 6A and 6B), indicating that production of hNIS/tdTomato protein is dependent upon cAMP concentration. To test the possible use of in vivo application, cAMP-treated or untreated HeLa-hNIS/tdTomato cells were transplanted on the flank of a mouse. Tumor cells treated with 100 μM of cAMP showed a 1.92-fold increase in fluorescent signals (Fig 6C and 6D) compared to tumor cells without cAMP treatment. These results suggest that the NIS/tdTomato fusion gene is useful for evaluating alterations in the amount of hNIS both in vitro and in vivo.

## Conclusions

In this study, we were able to visualize the cellular localization of hNIS by hNIS/tdTomato fusion reporter. Our results demonstrated that the membrane translocation of NIS depends on the level of glycosylation. Particularly, we observed that the amount of I-uptake and membrane-localized NIS are closely related, and cAMP treatment increases the amount of glycosylated NIS as well as NIS production itself. Based on imaging analysis and functional studies, we found that glycosylation plays a critical role in the membrane translocation of hNIS and this results increased iodine uptake.

## Compliance with Ethical Standards

Ethical approval: Animal experiments using mice were performed in accordance with the Institutional Animal Care and Use Committee of Seoul National University Hospital guidelines.

## Supporting Information

S1 Fig Z-stack image of glycosylation-mediated membrane translocation of hNIS/tdTomato. Cross-sectional images of red fluorescence in HeLa-hNIS/tdTomato cells were analyzed to assess the localization of hNIS/tdTomato protein expression. Confocal microscope images were sectioned by acquiring Z-stacks at 1.5 mm-thick sections.
(TIF)
