# Supplementary figures and images for: Glycosylation of Sodium/Iodide Symporter (NIS) Regulates Its Membrane Translocation and Radioiodine Uptake
Source: PLoS One. 2015 Nov 23;10(11):e0142984. doi: 10.1371/journal.pone.0142984 (PMC4658105; doi:10.1371/journal.pone.0142984)

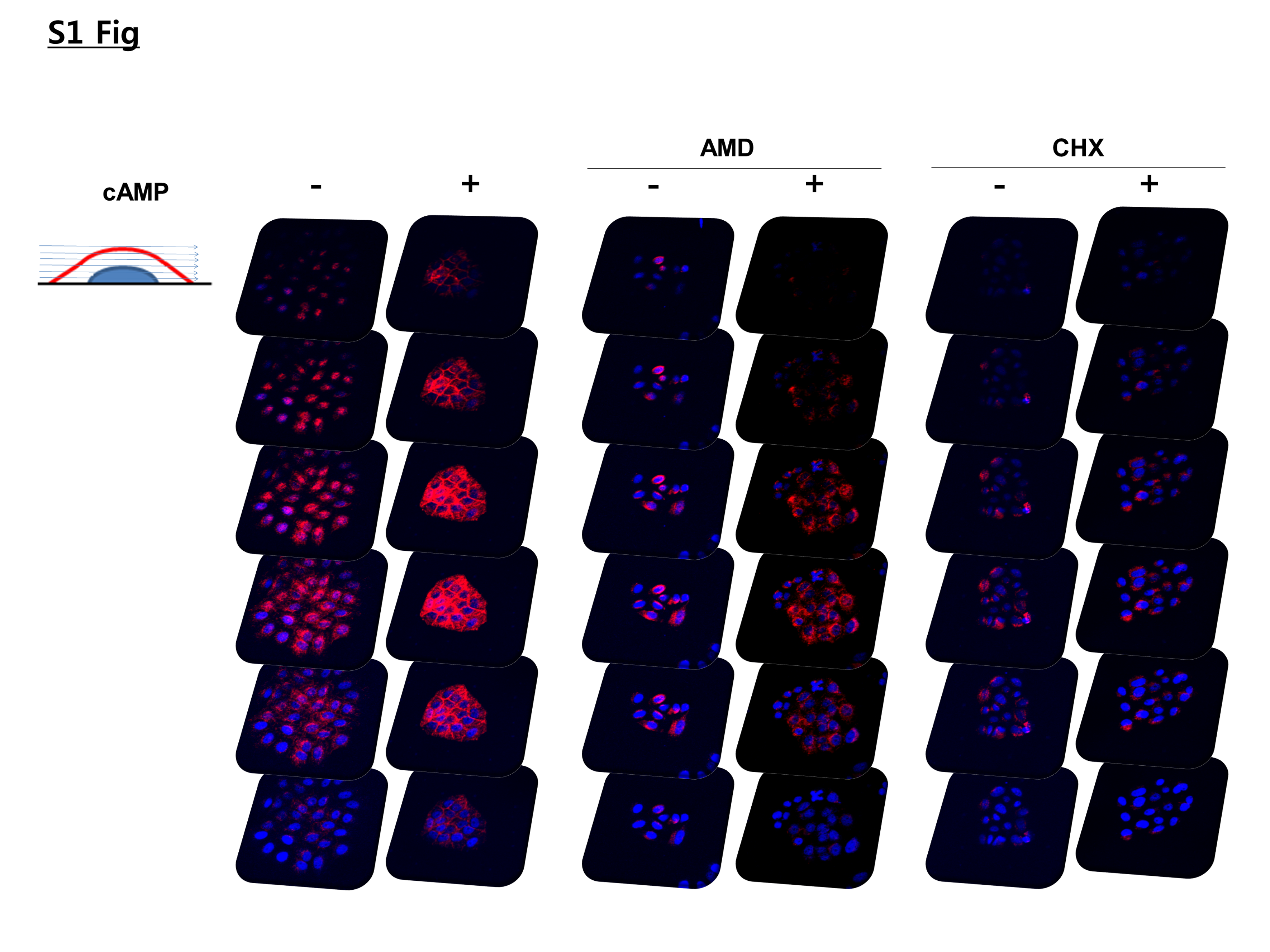

Supplement: S1 Fig — Cross-sectional images of red fluorescence in HeLa-hNIS/tdTomato cells were analyzed to assess the localization of hNIS/tdTomato protein expression. Confocal microscope images were sectioned by acquiring Z-stacks at 1.5 mm-thick sections. (TIF) [file pone.0142984.s001.tif]
